# Supplementary material for: Gauge equivariant convolutional neural networks for diffusion MRI
Source: Sci Rep. 2025 Mar 20;15:9631. doi: 10.1038/s41598-025-93033-1 (PMC11926199; doi:10.1038/s41598-025-93033-1)
Supplement: Supplementary file 1 — Supplementary Information. [file 41598_2025_93033_MOESM1_ESM.pdf]

1 Appendix

1.1 Models

Overall we trained four different kinds of architectures. In Figure 3 we have shown two architectures; the base model containing just 3d convolutions (3d), and the model which has a 3d convolution block followed by a gauge equivariant block (3d $\rightarrow$ Gauge). We also explored two other models; one which consisted of only a gauge equivariant block (Gauge) and one in which the gauge equivariant block preceded the 3d block (Gauge $\rightarrow$ 3d). In the Gauge model there are no inter-voxel interactions and all voxels are moved to the batch dimension. In this model there are four layers with 32 filters each. In the Gauge $\rightarrow$ 3d model, the Gauge model is followed by three layers of 3d convolutions with 48 filters each. Here the icosahedron points are moved to batch dimension when passed to the 3d convolutions block; so there is inter-voxel interactions but only for the same gradient direction. There is no T_1 or T_2 images in the input for the Gauge and Gauge $\rightarrow$ 3d models. Table 1 shows the time and memory consumption of each of these models.

We also test for changes to the input gradient directions, we define three types 1) Regular: these are the first six gradient directions as shown in above sections, 2) Rotate: here we take three random Euler angles per test subject and perform a 3d rotation of the gradient directions taken in the Regular type, 3) Random: here for each test subject we randomly pick six unique directions. In Table 2 we have summarized the accuracy metrics for ΔFA and $\Delta\theta$ for each model and the inputs, and for each type modification to the gradient directions. In Figure 1 the rows show FA and V_1 for a subject for each type of modification to the gradient directions, the columns show the inputs, each type of model and the ground truth.

Time elapsed and memory consumption

Model	#-Subs	Time(hrs)/Epoch	Memory(GB)/Batch
3d (base)	40	0.24	0.27
3d $\rightarrow$ Gauge	15	3.86	18.88
Gauge	15	3.66	12.70
Gauge $\rightarrow$ 3d	15	3.77	12.72

Table 1: This table shows the memory consumption per batch and time elapsed per epoch for each model.

Accuracy metrics under gradient vector changes

Model	#-Subs	Regular		Rotate		Random	
		ΔFA	Δθ	ΔFA	Δθ	ΔFA	Δθ
Inputs	-	0.277, 0.024	37.76, 1.30	0.277, 0.024	37.76, 1.30	0.451, 0.227	44.00, 7.73
3d (base)	40	0.110, 0.008	24.66, 1.67	0.110, 0.008	57.99, 8.41	0.116, 0.008	52.76, 5.44
3d $\rightarrow$ Gauge	15	0.053, 0.003	20.28, 1.33	0.056, 0.004	22.87, 5.47	0.092, 0.001	38.07, 3.27
Gauge	15	0.096, 0.003	31.04, 1.28	0.101, 0.003	33.26, 3.40	0.103, 0.001	33.38, 2.35
Gauge $\rightarrow$ 3d	15	0.073, 0.003	28.19, 1.32	0.081, 0.003	33.08, 3.41	0.083, 0.008	32.40, 2.34

Table 2: This table shows the effect on accuracy metrics in the format (mean, standard deviation) of changing the gradient vector sampling. ‘Regular’ denotes the first six vectors (presented in main text), ‘Rotate’ means each subject’s first six vectors were rotated by random Euler angles, and ‘Random’ means for each subject random six directions were chosen. These random Euler angles and directions are different for each subject. The rows show the inputs and the different models used.

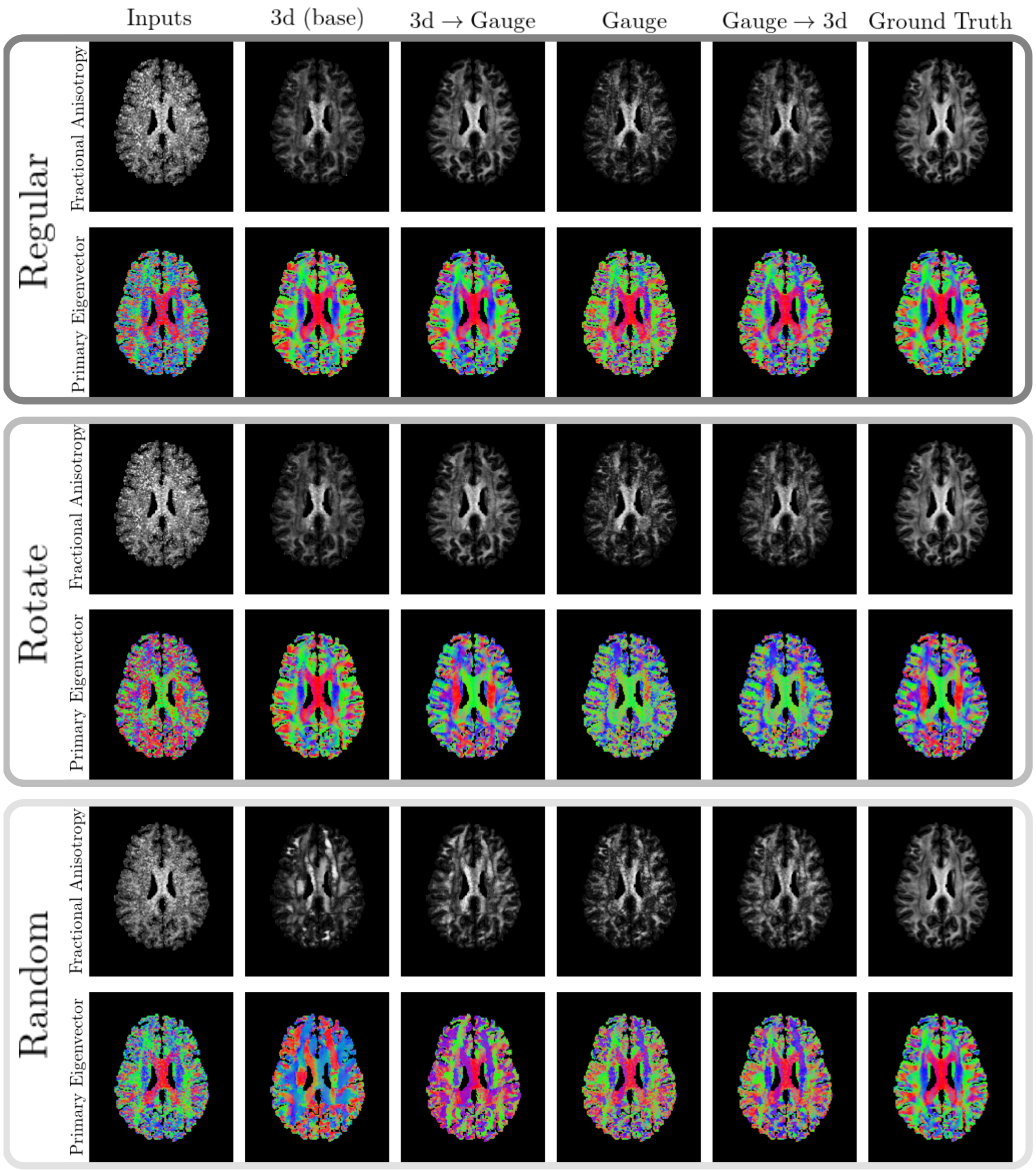

Figure 1: The rows in the figure show for one subject the FA and the primary eigenvector for each type of modification to the input gradient vectors. The columns show inputs, the different models, and the ground truth.

Accuracy metrics in ROIs

ROI	ΔFA			$\Delta\theta$		
	Inputs	Base	3d $\rightarrow$ Gauge	Inputs	Base	3d $\rightarrow$ Gauge
Left-Cerebral-White-Matter	0.30, 0.04	0.12, 0.02	0.07, 0.01	36.96, 3.55	25.44, 5.45	19.25, 2.93
Left-Lateral-Ventricle	0.29, 0.02	0.12, 0.02	0.03, 0.00	45.44, 2.52	40.84, 3.80	35.54, 4.32
Left-Inf-Lat-Vent	0.29, 0.03	0.06, 0.01	0.04, 0.01	42.56, 4.24	38.70, 6.03	33.26, 4.21
Left-Cerebellum-White-Matter	0.28, 0.03	0.17, 0.02	0.07, 0.01	35.36, 2.63	19.91, 2.44	16.18, 1.47
Left-Cerebellum-Cortex	0.30, 0.03	0.09, 0.01	0.06, 0.01	41.19, 2.98	26.45, 3.40	21.63, 1.96
Left-Thalamus-Proper	0.37, 0.03	0.14, 0.01	0.08, 0.01	40.35, 1.90	24.92, 2.58	20.77, 2.24
Left-Caudate	0.44, 0.04	0.08, 0.01	0.06, 0.01	45.93, 1.58	35.40, 2.90	30.77, 2.76
Left-Putamen	0.44, 0.04	0.09, 0.01	0.06, 0.01	44.15, 1.90	32.08, 3.19	28.73, 2.32
Left-Pallidum	0.54, 0.05	0.12, 0.03	0.10, 0.04	48.37, 1.69	39.85, 3.86	35.86, 3.31
3rd-Ventricle	0.30, 0.03	0.09, 0.02	0.07, 0.01	42.57, 2.77	34.26, 5.08	34.53, 3.07
4th-Ventricle	0.32, 0.02	0.13, 0.02	0.04, 0.01	48.08, 1.83	41.45, 2.41	35.25, 3.10
Brain-Stem	0.31, 0.03	0.15, 0.02	0.08, 0.00	37.51, 2.16	22.15, 2.48	17.83, 2.02
Left-Hippocampus	0.35, 0.03	0.07, 0.01	0.06, 0.00	43.83, 1.51	35.71, 3.06	32.00, 2.42
Left-Amygdala	0.35, 0.04	0.08, 0.01	0.06, 0.01	43.11, 2.14	36.61, 4.59	33.24, 2.95
CSF	0.38, 0.04	0.14, 0.03	0.06, 0.01	48.78, 2.97	41.82, 3.90	34.97, 3.63
Left-Accumbens-area	0.42, 0.05	0.08, 0.01	0.06, 0.01	43.93, 2.11	30.64, 3.17	26.68, 2.91
Left-VentralDC	0.33, 0.03	0.16, 0.02	0.09, 0.01	35.16, 2.08	21.28, 2.30	17.07, 2.06
Left-vessel	0.31, 0.08	0.08, 0.02	0.06, 0.01	38.30, 5.15	29.44, 5.36	27.81, 7.00
Left-choroid-plexus	0.32, 0.03	0.10, 0.01	0.05, 0.01	44.39, 2.18	38.28, 3.18	32.34, 3.61
Right-Cerebral-White-Matter	0.28, 0.04	0.13, 0.02	0.08, 0.01	34.24, 4.26	22.36, 4.01	17.64, 2.51
Right-Lateral-Ventricle	0.30, 0.02	0.13, 0.02	0.03, 0.00	46.09, 2.27	42.15, 3.42	37.42, 3.68
Right-Inf-Lat-Vent	0.29, 0.04	0.06, 0.01	0.05, 0.01	42.79, 5.21	35.84, 6.03	32.03, 6.14
Right-Cerebellum-White-Matter	0.29, 0.03	0.16, 0.02	0.07, 0.00	33.73, 2.83	19.46, 2.87	16.51, 1.49
Right-Cerebellum-Cortex	0.31, 0.04	0.09, 0.01	0.07, 0.01	39.90, 2.42	27.97, 3.21	23.21, 1.98
Right-Thalamus-Proper	0.40, 0.04	0.12, 0.01	0.08, 0.01	42.14, 2.18	26.39, 3.15	21.61, 2.55
Right-Caudate	0.41, 0.03	0.08, 0.01	0.06, 0.01	44.70, 2.05	32.72, 3.35	28.45, 3.00
Right-Putamen	0.43, 0.04	0.10, 0.01	0.07, 0.01	44.12, 2.13	32.42, 4.46	28.73, 3.17
Right-Pallidum	0.58, 0.04	0.13, 0.02	0.12, 0.03	50.14, 1.49	42.23, 4.07	37.04, 3.69
Right-Hippocampus	0.35, 0.04	0.08, 0.01	0.06, 0.00	43.09, 1.74	35.85, 3.21	32.25, 2.45
Right-Amygdala	0.33, 0.03	0.09, 0.02	0.07, 0.01	41.50, 2.47	36.80, 3.90	31.17, 3.96
Right-Accumbens-area	0.39, 0.04	0.08, 0.01	0.06, 0.01	43.55, 2.13	33.84, 4.57	30.24, 4.13
Right-VentralDC	0.33, 0.03	0.17, 0.02	0.09, 0.01	37.99, 2.39	22.15, 2.96	17.68, 1.78
Right-vessel	0.31, 0.06	0.09, 0.02	0.07, 0.01	39.54, 6.37	22.88, 5.21	18.83, 4.28
Right-choroid-plexus	0.31, 0.03	0.10, 0.02	0.05, 0.01	43.32, 2.24	37.06, 3.36	33.05, 3.20
WM-hypointensities	0.26, 0.09	0.07, 0.03	0.04, 0.01	41.19, 6.40	27.79, 7.91	25.30, 10.06
Optic-Chiasm	0.31, 0.09	0.15, 0.03	0.09, 0.02	33.51, 4.50	28.23, 6.26	20.68, 5.06
CC_Posterior	0.18, 0.02	0.23, 0.04	0.07, 0.01	24.13, 2.18	7.41, 1.05	6.76, 1.11
CC_Mid_Posterior	0.22, 0.03	0.17, 0.03	0.06, 0.01	26.39, 4.68	9.52, 2.03	8.22, 1.90
CC_Central	0.22, 0.03	0.17, 0.04	0.07, 0.01	23.51, 5.13	9.37, 3.13	7.96, 1.75
CC_Mid_Anterior	0.22, 0.03	0.18, 0.03	0.07, 0.01	27.63, 3.45	10.93, 2.28	9.64, 1.31
CC_Anterior	0.21, 0.02	0.24, 0.03	0.08, 0.01	30.39, 4.11	10.93, 2.16	9.09, 1.86

Table 3: This table shows the accuracy metrics in different brain areas. We compute the mean ΔFA and mean $\Delta\theta$ over the voxels in each brain region. Each column contains the mean and standard deviation of these quantities respectively, which is computed over all 25 test subjects. The ΔFA and $\Delta\theta$ columns are split into the inputs, base model, and the 3d $\rightarrow$ Gauge architecture.

1.2 Brain regions

We computed the accuracy metrics for each ROI in the brain; the calculation is performed in the same manner as **??** except the average to compute ΔFA and $\Delta\theta$ is computed over the voxels in each brain ROI rather than all voxels in the brain mask. These quantities are then averaged over all 25 test subjects. This is done for the two models shown in **??**,

and results are shown in Table 3, each entry in the column contains two, comma separated, numbers which are the mean and standard deviation respectively.
